# Factors Influencing the Smartphone Usage Behavior of Pedestrians: Observational Study on “Spanish Smombies”

**DOI:** 10.2196/19350

**Published:** 2020-08-14

**Authors:** César Fernández, María Asunción Vicente, Irene Carrillo, Mercedes Guilabert, José Joaquín Mira

**Affiliations:** 1 Telematics Engineering Area Miguel Hernández University Elche Spain; 2 Health Psychology Department Miguel Hernández University Elche Spain; 3 Foundation for the Promotion of Health and Biomedical Research of Valencia Region Alicante Spain; 4 Alicante-Sant Joan Health District Alicante Spain

**Keywords:** smartphone addiction, smartphone overuse, smombies, pedestrian safety, mobile phone

## Abstract

**Background:**

Smartphone addiction has become a reality accepted by all. Some previous studies have shown that the use of smartphones on public roads while walking is very common among the young population. The term “smombie” or smartphone zombie has been coined for this behavior. Such behavior causes a reduction in the attention given to other pedestrians and drivers and may result in accidents or collisions. However, there are no precise data about how many people use the phone while they are walking on the street. Smartphone usage habits are evolving rapidly, and more in-depth information is required, particularly about how users interact with their devices while walking: traditional phone conversations (phone close to the ear), voice chats (phone in front of the head), waiting for notifications (phone in hand), text chats (user touching the screen), etc. This in-depth information may be useful for carrying out specific preventive actions in both the education field (raising awareness about the risks) and in the infrastructure field (redesigning the cities to increase safety).

**Objective:**

This study aimed to gather information about pedestrians’ smartphone usage and to identify population groups wherein interventions should be focused to prevent accidents. The main hypothesis was that gender, age, and city area can significantly influence the smartphone usage of the pedestrians while walking.

**Methods:**

An observational study of pedestrians in the street was carried out in Elche, a medium-sized Spanish city of 230,000 inhabitants. The following data were gathered: gender, age group, location, and type of smartphone interaction. A specific smartphone app was developed to acquire data with high reliability. The statistical significance of each variable was evaluated using chi-squared tests, and Cramér’s V statistic was used to measure the effect sizes. Observer agreement was checked by the Cohen kappa analysis.

**Results:**

The behavior of 3301 pedestrians was analyzed, of which 1770 (53.6%) were females. As expected, the effect of the main variables studied was statistically significant, although with a small effect size: gender (*P*<.001, V=0.12), age (*P*<.001, V=0.18), and city area (*P*<.001, V=0.16). The phone in hand or “holding” behavior was particularly dependent on gender for all age groups (*P*<.001, V=0.09) and to a greater extent in young people (*P*<.001, V=0.16). Approximately 39.7% (222/559) of the young women observed showed “holding” or “smombie” behavior, and they comprised the highest proportion among all age and gender groups.

**Conclusions:**

An in-depth analysis of smartphone usage while walking revealed that certain population groups (especially young women) have a high risk of being involved in accidents due to smartphone usage. Interventions aimed at reducing the risk of falls and collisions should be focused in these groups.

## Introduction

### Use of Mobile Devices and Smartphones

Since the emergence of mobile technology, the use of mobile devices and services has continued to increase progressively and at different rates in both developed and developing countries [1]. By the end of 2018, more than 5 billion people around the world subscribed to mobile services, accounting for 67% of the global population, and this figure is expected to reach 71% by 2025 [2]. In Spain, the penetration rate of mobile devices is 98%, with most of them (80%) being smartphones [1]. In both cases, the penetration rate in Spain is above the European average (85% and 72%, respectively) [2]. There are differences in the penetration rate of smartphones by age; 95% of the Spaniards younger than 35 years own a smartphone, while only 60% of those older than 50 years own a smartphone—a trend that is widespread worldwide [1]. Further, 3.6 billion people are connected to the mobile internet, with 67% of the global connections occurring through smartphones [2,3].

These data show the globalized presence of mobile devices, which is inevitably linked to their increasing use in time and place. In 2018, users around the world spent an average of 800 hours per year on their smartphones. In Europe, the average time spent on smartphones is 3 hours daily and between 14 and 43 hours weekly. The most common activities among Europeans include (in this order) emailing, social networking, instant messaging, searching, reading, and gaming [4]. Given these high levels of dedication, it is not surprising that the use of smartphones overlaps with the execution of other activities (multitasking) with variable attention requirements such as watching television, eating, dressing, working, or walking, including frequent checks and alternating periods of activity to attend to possible notifications [5].

### Problematic Smartphone Use

Despite the many benefits of smartphones, their unlimited use can lead to what is known as problematic smartphone use, which is related to the discomfort associated with “unsubstantiated or behavioral addictions” (eg, anxiety when the device is not accessible) [6]. According to a recent meta-analysis by Sohn et al [7] which included 41,871 children and young people, the median prevalence of problematic smartphone use was 23.3%. Age (17-19 years) and female gender were the risk factors for the development of problematic smartphone use, although in the case of the latter, the results are not conclusive. Problematic smartphone use is associated with higher odds of experiencing depression, anxiety, perceived stress, and a decrease in sleep quality. One of the problematic uses can arise, for example, when crossing a street; this is a complex exercise with a relatively high demand for perceptive and cognitive capacity. Even for those pedestrians who can successfully integrate the required information under normal circumstances, the distraction of holding a smartphone can interfere with the decision-making process at many points. Pedestrians may be unaware of important auditory or visual information, make incorrect judgments about speed (especially when multiple lanes or vehicles are involved), incorrectly attribute driver intent, or misjudge their ability to cross in a given gap. Distraction, therefore, has the potential to exacerbate the risk of a collision for pedestrians [8]. In a study [9] that aimed to explore the effect of gender on the use of smartphones while walking, a modest gender bias was observed, with walking behavior with the smartphone more frequent among women than men. Another effect observed was that when couples of the opposite sex walked together, the use of the smartphone was decreased. Some of the reasons for people to walk with smartphones in their hands could be the social pressure to be available, security concerns (reduced risk of theft), psychological dependence (anxiety over separation from the smartphone), or for display as a status symbol.

### Smombies

The term “smombie” (smartphone-zombie) [10,11] or “phone walker” [9] has been coined as a result of increasingly frequent behavior involving the use of smartphones while walking on public roads [9]. This concept refers to the pedestrian who uses a smartphone while walking, with the physical or cognitive consequences that this type of behavior may have. The effects on physical health are the carrying out of behaviors that may endanger the pedestrian or other people who are circulating at that moment—mainly the lack of safety [12,13]. The other effects may be directly related to the way one walks and one’s direct involvement in a traffic accident compared to people who do not use their smartphones while walking [13]. At the cognitive level, the lack of attention while walking and using the smartphone implies a lack of recognition of the other pedestrians, lower cognitive capacity, and greater attention deficit [14]. It is arguable whether smombie behavior represents a form of problematic smartphone use in itself or not. However, there is no doubt that this behavior represents a safety risk. It seems that this phenomenon is increasingly being studied in different countries and contexts [15,16] and evidences on the effects and consequences of this pattern of behavior are increasing [17,18], because of which this study was carried out. The goal of this study was to gather the information that helps us measure and understand the smombie behavior and to identify specific groups that may require special attention to reduce the risk of accidents.

## Methods

### Study Design

An observational study of the behavior of pedestrians with their smartphones was carried out in Elche, Spain, by a multidisciplinary research group composed mainly of behavioral scientists and smartphone engineers from Miguel Hernández University. It was executed from April 2019 until November 2019 as a project called “CountingSmombies.” This study was registered and validated by the ethics committee of the Miguel Hernández University with the research code COIR:AUT.DISP.CFP.01.19. Direct measurements by an observer was the method used to register the behavior of the pedestrians with their smartphones while they were walking on the street. The behavior of the pedestrians with their smartphones was categorized into the following 5 classes, which is ordered from lower to higher use of the smartphone.

1. NOT VISIBLE: The pedestrian does not visibly carry or use his/her smartphone.
2. TALKING: The pedestrian is talking on the phone in the traditional way, that is, the smartphone is held in the hand and close to the ear and mouth of the talking subject.
3. HEADPHONES: The pedestrian is wearing headphones visibly and these are supposed to be connected to a smartphone.
4. HOLDING: The pedestrian holds the smartphone in one of his/her hands while walking but is not looking directly at it.
5. SMOMBIE: The pedestrian holds the smartphone in his/her hand while walking and interacts with the screen by either staring at it or typing or talking toward the screen when the audio is sent or during videoconferencing.

These 5 categories were selected according to that reported in recent studies [19,20] and by considering 2 main factors: first, they were easily distinguishable by the observers and second, they represented different attitudes toward smartphone usage while walking. The ordering below reflects, what we considered, an increasing risk of behavioral addiction or attention loss:

1. Talking in a traditional way was considered as less invasive smartphone usage, as only one ear is involved.
2. Using headphones was associated with higher attention loss, with both ears involved.
3. Holding the smartphone in our hands, although apparently not causing attention loss, may reflect a psychological dependence, a need to be aware of incoming notifications, and can be part of an alternation of smombie-holding periods. That is why we considered such behavior almost on top of the problematic smartphone use list.

**Figure 1:**
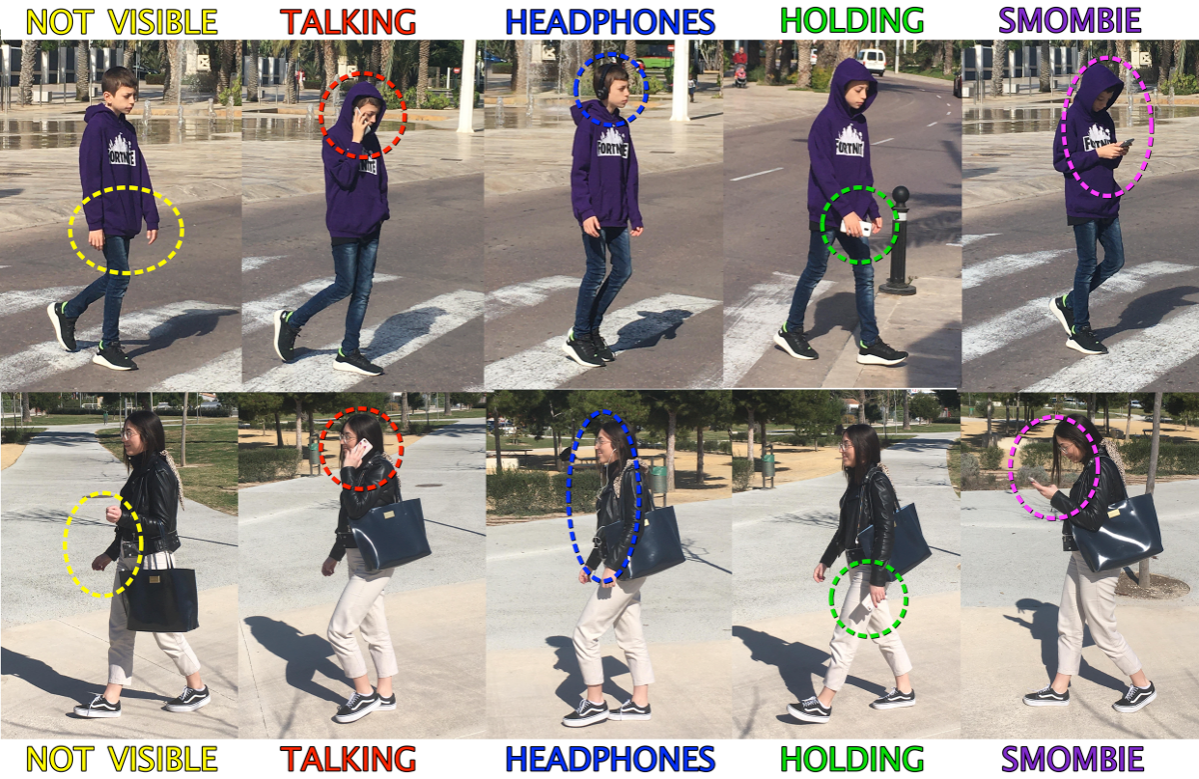
The 5 types of smartphone usage behaviors observed in street pedestrians. From left to right (lower to higher use of smartphone): smartphone "not visible," "talking" on a smartphone, using "headphones," "holding" a smartphone, and "smombie".

Figure 1 illustrates the 5 types of smartphone behaviors observed in street pedestrians. The observer also registered the gender and the approximate age of the pedestrian according to the perceived appearance (the interobserver agreement was validated through a Cohen kappa analysis in a simultaneous session with 2 observers and with N=100). Only 2 classes were used in the gender category: male and female. For the age category, 4 classes were used: (1) below 18 years (10-18 years), teenagers; (2) 18-35 years, young people; (3) 35-65 years, adults; (4) over 65 years, older people. Data were stored by using a quick annotation app that allowed saving the data of each of the performed experiments.

### Pedestrian Observation Procedure

**Figure 2:**
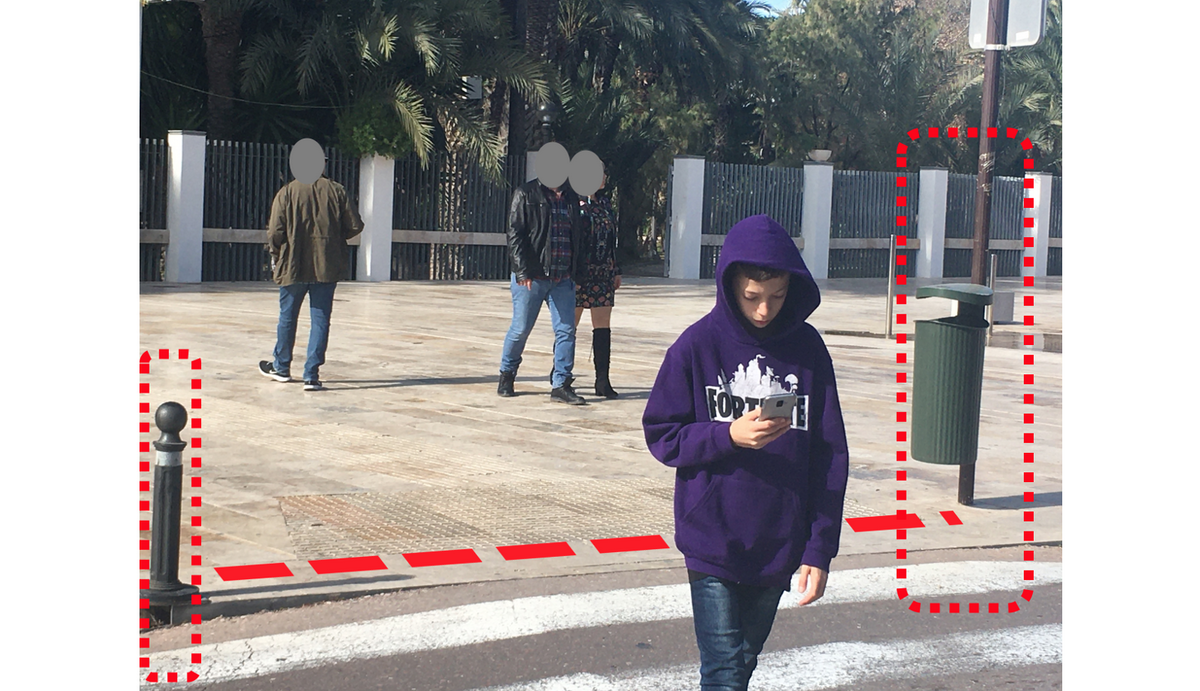
Defining a visual crossing line between 2 points on the street, for example, the bollard and the street litter bin, to not introduce bias and to count only the pedestrians moving in one of the two directions between the crossing line.

A pedestrian observation procedure was designed with 2 modalities: sitting observer and moving (walking) observer. Sitting observers stayed at a specific location and registered pedestrians in their field of vision. Moving observers walked through a predetermined path and registered pedestrians walking in the opposite direction. When the observer was walking, it was easy to avoid biases in the selection of the pedestrians; only those who randomly cross with the observer were recorded. When the observer was sitting, a stricter protocol was needed; there were many pedestrians around and only some of them may catch the observer’s attention. According to our protocol, only those pedestrians who pass through a predefined crossing line were recorded. The crossing line was defined between 2 points on the street, for example, 2 trees, 2 bollards, or the sides of a shop window (Figure 2). Besides, the pedestrians walking through the crossing line could walk in either direction; therefore, a predefined direction was established and only pedestrians walking in such a direction were recorded.

A pedestrian eligibility list was given to the observers to homogenize their recordings (Textbox 1). Pedestrians were included if they were walking alone or in a group. However, groups of over 4 people were excluded to avoid counting errors. It was decided to include people who run but not those who use vehicles such as bicycles and wheel or electric scooters. People in wheelchairs (and their porters) and those who push a baby carriage were included. A common behavior that was observed among the pedestrians was that they took their smartphones from their pockets or purses and stopped or even sat or leaned on an urban structure to use the smartphone more slowly. When this occurred during an observational experiment, this subject was not counted, as this situation fell outside the established categories (ie, the subject was not walking).

Pedestrians who used a wristband or watch were also excluded because it was difficult to assess their category; the observer cannot distinguish whether they were wearing a traditional watch or a smartwatch. In the first case, the pedestrian may just be looking at the time; in the second case, the pedestrian may be accessing a secondary smartphone screen. Globally, the eligibility list introduced a bias in the measures: the actual proportions of the smombies may have been higher than that accounted for (eg, smartwatches are excluded). However, our goal was to establish very clear criteria so that different observers could obtain homogeneous measures.

**Figure 3:**
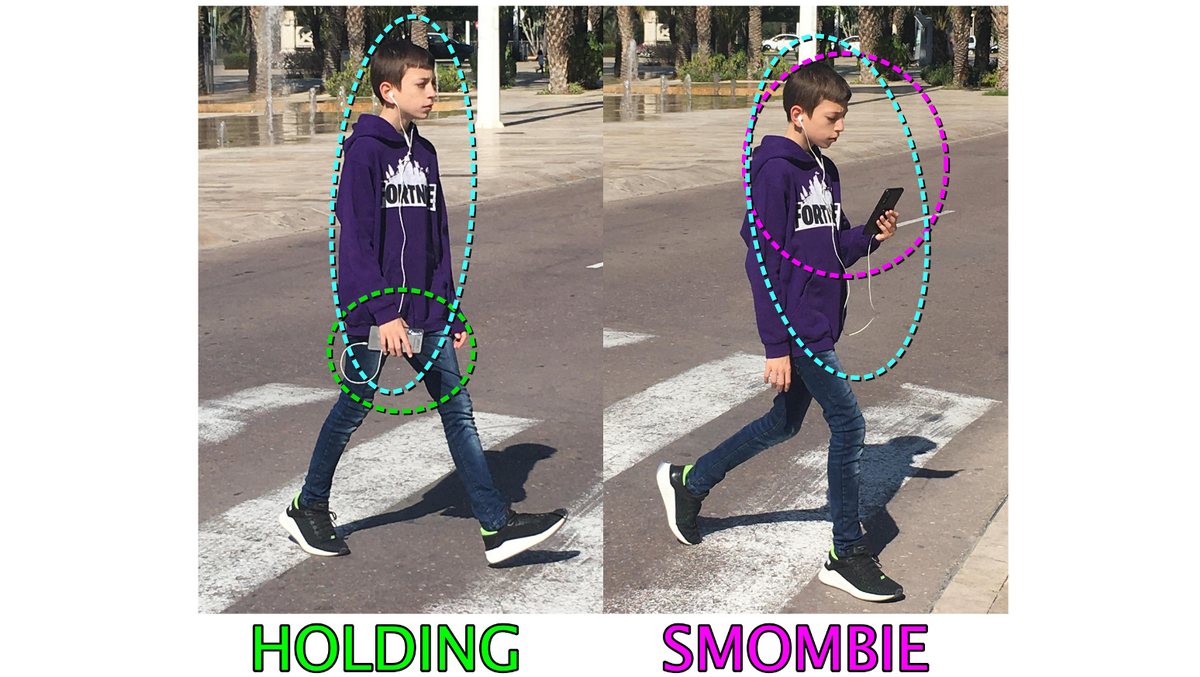
In pedestrians with mixed behavior, higher problematic smartphone usage was selected as the pedestrian behavior.

The pedestrian could simultaneously present 2 established behaviors (or 2 mixed categories), for example, using headphones and looking at the smartphone screen. In these cases, the observer should select the behavior where the problematic use of smartphone is higher. Figure 3 shows 2 possible mixed categories: using headphones and holding the smartphone simultaneously was categorized as “holding” class and using headphones and interacting with the smartphone was categorized as “smombie” class.

**Eligibility criteria for the pedestrians.**

**Pedestrians included in the observational data**

- people walking alone or in a group of 4 people or less
- people running or jogging
- people in wheelchairs and their companions
- people walking with their dogs
- people carrying baby carriages

**Pedestrians excluded from the observational data**

- groups of more than 4 people
- bike or scooter travelers
- those who stopped walking and stood while using the smartphone
- people who were doing a job (eg, postmen, carriers, gardeners, waiters)
- those who checked a wristband or wristwatch
- any other situation not considered above

### Observation Sessions

Data were registered during 43 observation sessions. An observation session requires the selection of a placement (sitting observer) or path (moving observer) as well as the selection of the date and time for the recording. The session duration was not predetermined, and the number of pedestrians registered per session were not predetermined. The quick annotation app allowed continuous recording without limits in time or number of registers.

Concerning date and time, observation sessions were performed from July 2019 to November 2019 during working days and during rush hours (except for the *Pokemon Go Community Day*, an extra observation session). Two periods of the day were considered: midday rush hour (noon to 2 PM) and evening rush hour (7 PM to 9:30 PM). According to the Spanish schedule, since most people leave work or school during these periods, there are plenty of people and activities on the street.

An extra observation session was performed on Saturday, October 12, 2019 on the *Pokemon Go Community Day* [21], a worldwide monthly event wherein *Pokemon Go* players get together to look for special game items (specific *Pokemons* that appear with high frequency at certain time lapses and city areas). This resulted in a notable increase in the number of *Pokemon Go* players on the street and consequently, in the number of smombies observed. The goal was to analyze the differences with the remaining sessions.

Concerning the location, all the observation sessions were performed in Elche (medium-sized Spanish city, 230,000 inhabitants), where 3 different scenarios were selected: city center, residential areas (with large avenues frequented by runners and hikers), and different areas of the University campus. These scenarios covered a wide variety of city inhabitants and situations such as people walking to or from work in the city center, people in their leisure time in residential areas, and students in the University campus. The special observation session corresponding to the *Pokemon Go Community Day* was performed in the city center.

### Quick Annotation App

**Figure 4:**
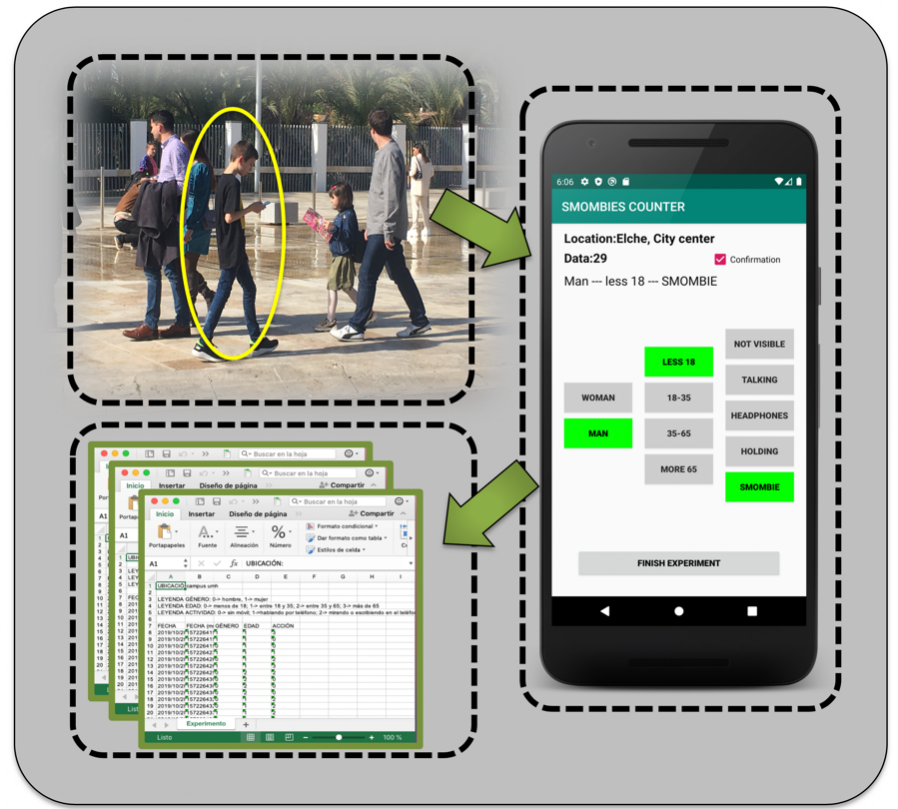
A screenshot of the quick annotation app. Data were stored in the csv format and processed in Matlab and R software.

The main screen of the quick annotation app is shown in Figure 4. The purpose of using the app was to be able to quickly and efficiently save the measurement made by the observer on a spreadsheet. Acquiring data was fast. It just required 3 taps per subject to register: gender button, age button, and behavior button. The selected options light up in green for a short time to provide visible feedback to the observer. When an observational session ends, simply by clicking on the “Finish Experiment” button, the data were saved on a spreadsheet and stored for further processing and analysis.

### Statistical Methods

Each observation session was stored in csv files, which were processed with Matlab (version R2019a). All statistical analyses were performed using R software (version 3.6.2). All variables were considered categorical (including age, which was grouped into 4 age ranges). According to this, the chi-square test was used to evaluate the statistical significance of each variable, while effect sizes were measured using Cramér’s V statistic. Observer agreement was checked by the Cohen kappa analysis.

## Results

### Descriptive Statistics

A total of 3301 pedestrians were registered, of which 1770 (53.6%) pedestrians were women. According to the data from this observational study, 29.7% (982/3301) of the observed pedestrians were walking using a smartphone (“talking”, “headphones”, “holding”, and “smombies” classes) during rush hours on the working days in this city. The descriptive statistics of the study are shown in Table 1. To validate the study tool (clearly exclusive categories and the quick annotation app performance), 2 judges (sitting observers) performed a Cohen kappa analysis in a simultaneous session with a sample population of 100. In all categories, the degree of acceptance of the judges was high: gender of the pedestrians (=1; *P*<.001), age group (=0.703; *P*<.001), and behavior with the smartphone (=0.953; *P*<.001). Multimedia Appendix 1 shows the confusion matrices obtained in the Cohen kappa analysis for each category of the study.

**Table 1:** Demographic characteristics of the observed pedestrians in the study (N=3301).

Characteristics		Value	
**Gender, n (%)**			
	Male		1531 (46.4)
	Female		1770 (53.6)
**Age (years), n (%)**			
	Teenagers (10-18 years)		260 (7.9)
	Young people (18-35 years)		1179 (35.7)
	Adults (35-65 years)		1338 (40.5)
	Older people (>65 years)		524 (15.9)
**Smartphone use, n (%)**			
	Not visible		2319 (70.3)
	Talking		141 (4.3)
	Headphones		99 (3.0)
	Holding		381 (11.5)
	Smombie		361 (10.9)
**Scenarios, n (%)**			
	City center		2158 (65.4)
	Residential areas		623 (18.9)
	University campus		520 (15.7)

### Gender Influence

First, we analyzed the influence of the pedestrians’ gender on their smartphone usage behavior (Table 2). Overall, the influence was found to be statistically significant (*P*<.001), although with a small effect size (Cramér’s V=0.12).

**Figure 5:**
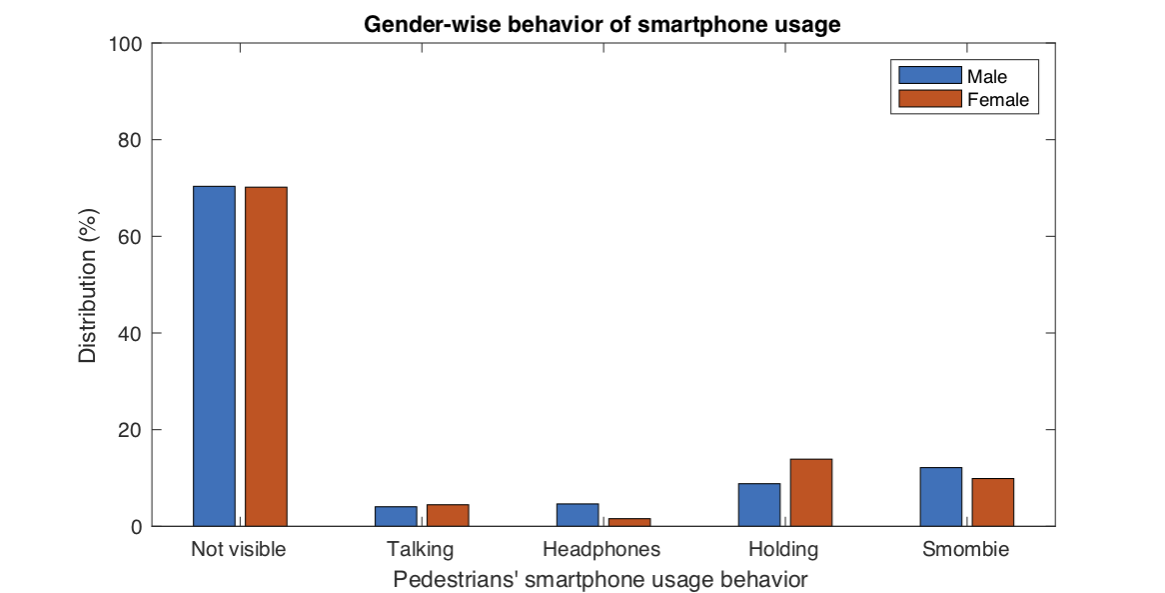
Smartphone usage behavior of pedestrians across different genders. This figure shows the observational results (%) from Table 2.

Table 2 shows the complete distribution of the data collected according to gender, as well as additional results: the “Headphones” and “Holding” behaviors were particularly influenced by gender. Women showed the “Holding” behavior (*P*<.001, V=0.09) to a greater extent, while men showed the “Headphones” behavior (*P*<.001, V=0.08) to a greater extent. For a better understanding of the results, Figure 5 shows the differences in the smartphone usage behavior according to gender.

**Table 2:** Influence of the pedestrians’ gender on smartphone usage behavior.

Behavior	Females, n=1770, n (%)	Males, n=1531, n (%)	*P* value	Cramér’s V
Not visible	1242 (70.2)	1077 (70.4)	.94	0.00
Talking	79 (4.4)	62 (4.1)	.62	0.01
Headphones	28 (1.6)	71 (4.6)	<.001	0.09
Holding	246 (13.9)	135(8.8)	<.001	0.08
Smombie	175 (9.9)	186 (12.1)	<.001	0.04

### Age Influence

Second, we analyzed the influence of the user’s age on their smartphone usage behavior. Overall, a statistically significant influence was also detected (*P*<.001), although the effect size was small (Cramér’s V=0.18). Table 3 shows the complete distribution according to age and additional results, which are consistent with the expectations. In this case, the behaviors most correlated with age were “Not visible” (ie, the pedestrian was not using the smartphone at all) (*P*<.001, V=0.30) and “Smombie” (*P*<.001, V=0.22). In the first case, the results showed that the use of the smartphone while walking was inversely related to age, and in the second case, an opposite behavior was observed.

**Table 3:** Influence of age on smartphone usage behavior in pedestrians.

Behavior	Teenagers^a^, n=260, n (%)	Young people^b^, n=1179, n (%)	Adults^c^, n=1338, n (%)	Older people^d^, n=524, n (%)	*P* value	Cramér’s V
Not visible	157 (60.4)	647 (54.9)	1037 (77.5)	478 (91.2)	<.001	0.30
Talking	6 (2.3)	67 (5.7)	58 (4.3)	10 (1.9)	.001	0.07
Headphones	16 (6.2)	55 (4.7)	24 (1.8)	4 (0.8)	<.001	0.10
Holding	43 (16.5)	182 (15.4)	133 (10.0)	23 (4.4)	<.001	0.13
Smombie	38 (14.6)	228 (19.3)	86 (6.4)	9 (1.7)	<.001	0.22

^a^*P*<.001; Cramér’s V=0.09.

^b^*P*<.001; Cramér’s V=0.26.

^c^*P*<.001; Cramér’s V=0.15.

^d^*P*<.001; Cramér’s V=0.20.

**Figure 6:**
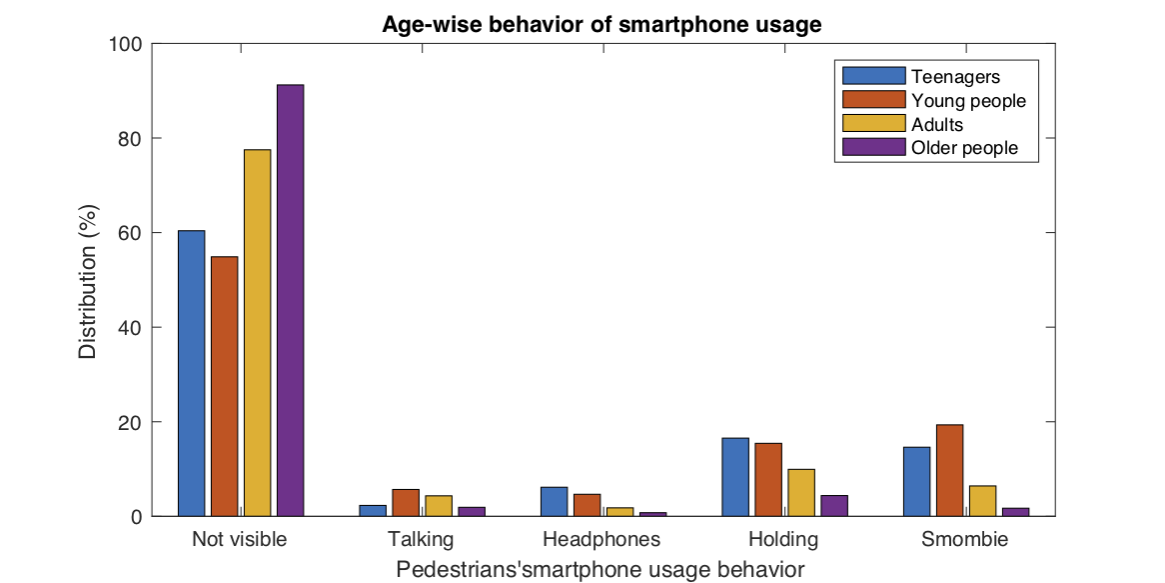
Smartphone usage behavior of pedestrians across different age ranges. This figure shows the observational results (%) from Table 3.

Adolescents and, to a greater extent, young people were the most likely to be in the “Smombie” category, while the older people were the least likely. Regarding the age ranges in which the pedestrians showed a different behavior from the average, the behavior of the young people (*P*<.001, V=0.26) and the older people (*P*<.001, V=0.20) was notable. Young pedestrians showed the highest values in the “smombie” and “talking” categories, while old pedestrians showed the highest value in the “not visible” category. Figure 6 shows the differences in the smartphone usage behavior according to age.

### Zone Influence

**Figure 7:**
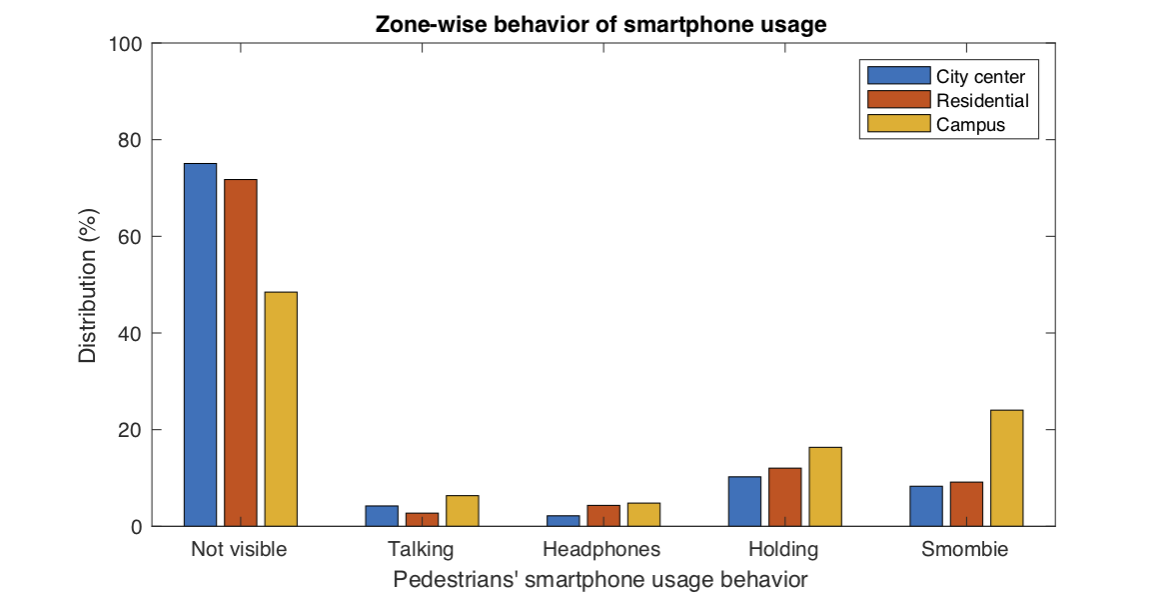
Smartphone usage behavior of pedestrians in different areas of a city. This figure shows the observational results (%) from Table 4.

We also analyzed whether different behaviors were associated with different zones or city areas (city center, residential areas, and University campus). Globally, the zone was statistically significant for behavior (*P*<.001), with a relatively small effect size (Cramér’s V=0.16). Table 4 shows the complete distribution and all the analyses performed. The behaviors more dependent on the zone were “Not visible vs other behaviors” (*P*<.001, V=0.20) and “smombie vs other behaviors” (*P*<.001, V=0.18). The zone that was clearly different from the others was the University campus (*P*<.001, V=0.22), where the median age of the pedestrians was different from that of the pedestrians in other city areas. In the University campus, the proportion of “smombies” was as high as 24.0% (125/520) and the proportion of people walking without using the phone at all was as low as 48.5% (252/520), which were very different from the data in other city areas. Figure 7 shows the differences in the smartphone usage behavior of the pedestrians according to the city area.

**Table 4:** Influence of the city area on pedestrian smartphone usage behavior.

Behavior	Campus,^a^ n=520, n (%)	City center,^b^ n=2158, n (%)	Residential,^c^ n=623, n (%)	*P* value	Cramér’s V
Not visible	252 (48.5)	1620 (75.1)	447 (71.8)	<.001	0.21
Talking	33 (6.4)	91 (4.2)	17 (2.7)	.01	0.05
Headphones	25 (4.8)	47 (2.2)	27 (4.3)	<.001	0.07
Holding	85 (16.3)	221 (10.2)	75 (12.0)	<.001	0.07
Smombie	125 (24.0)	179 (8.3)	57 (9.2)	<.001	0.18

^a^*P*<.001; Cramér’s V: 0.22.

^b^*P*<.001; Cramér’s V: 0.16.

^c^*P*=.02; Cramér’s V: 0.06.

### Gender and Age Influence

A deep analysis of the results allowed us to determine in which age range the behavior was most affected by gender. Table 5, Table 6, Table 7, and Table 8 show the grouped data, and we observed that the greatest differences in the behavior according to gender occurred in the young age range (*P*<.001, V=0.19). An additional analysis was carried out to specify the influence of gender on smartphone usage behavior in young people. The effect of gender on each of the behaviors (not visible, talking, headphones, holding, smombie) was studied for all age ranges. In particular, for the young age range (Table 6), the behavior most affected by gender was the “holding” behavior, which was much more common among women (*P*<.001, V=0.16).

**Figure 8:**
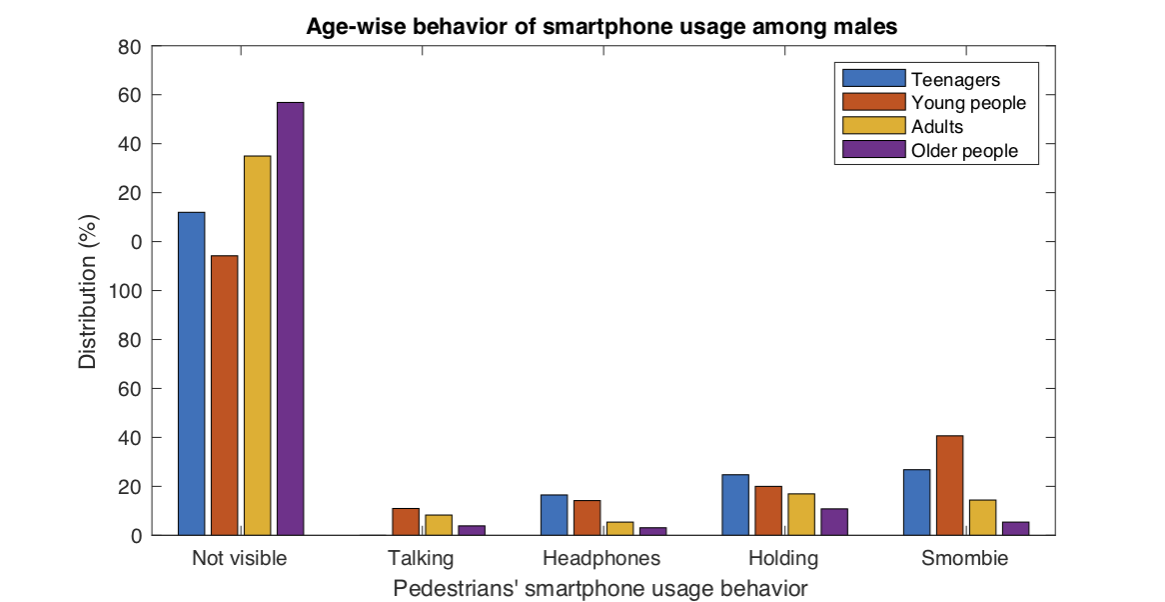
Smartphone usage behavior of males across different age ranges.

**Figure 9:**
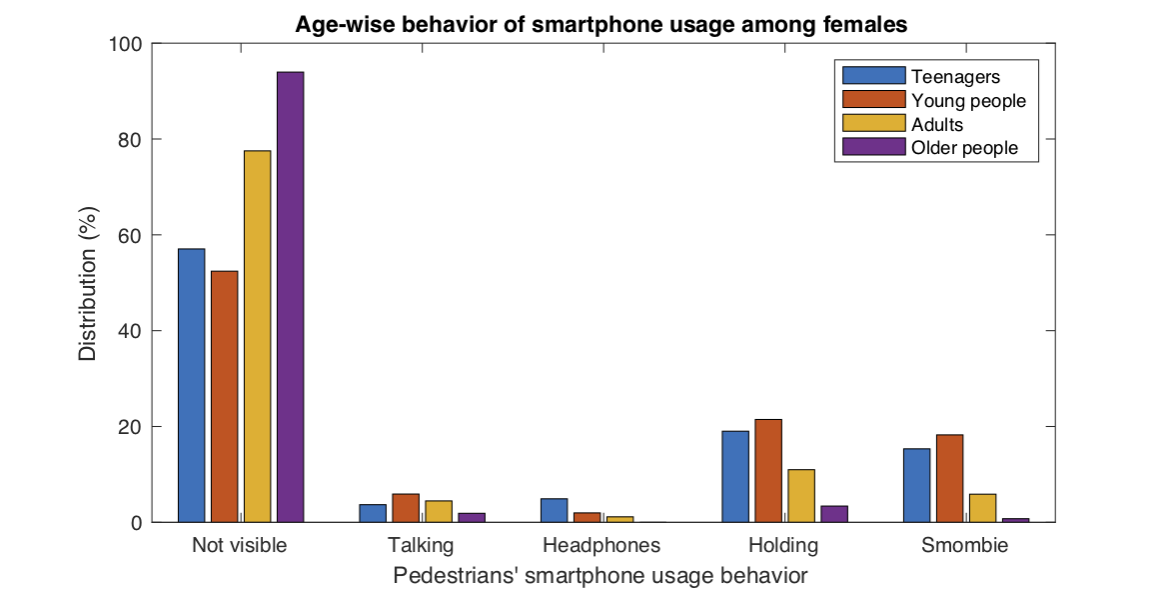
Smartphone usage behavior of females across different age ranges.

Figures 8-9 show graphically the combined effects of gender and age on smartphone usage behavior.

**Table 5:** Influence of gender on smartphone usage behavior in teenagers (n=260, globally *P*=.12, V=0.17).

Behavior	Females, n=163, n (%)	Males, n=97, n (%)	*P* value	Cramér’s V
Not visible	93 (57.1)	64 (66)	.19	0.080
Talking	6 (3.7)	0 (0)	.14	0.092
Headphones	8 (4.9)	8 (8)	.41	0.051
Holding	31 (19.0)	12 (12)	.22	0.076
Smombie	25 (15.3)	13 (13)	.81	0.015

**Table 6:** Influence of gender on smartphone usage behavior in young people (n=1179, globally *P*<.001, V=0.19).

Behavior	Females, n=559, n (%)	Males, n=620, n (%)	*P* value	Cramér’s V
Not visible	293 (52.4)	354 (57.1)	.12	0.045
Talking	33 (5.9)	34 (5.5)	.85	0.005
Headphones	11 (2.0)	44 (7.1)	<.001	0.117
Holding	120 (21.5)	62 (10.0)	<.001	0.156
Smombie	102 (18.2)	126 (20.3)	.41	0.024

**Table 7:** Influence of gender on smartphone usage behavior in adults (n=1338, globally *P*=.11, V=0.074).

Behavior	Females, n=783, n (%)	Males, n=555, n (%)	*P* value	Cramér’s V
Not visible	607 (77.5)	430 (77.5)	>.99	0.000
Talking	35 (4.5)	23 (4.1)	.88	0.004
Headphones	9 (1.1)	15 (2.7)	.06	0.052
Holding	86 (11.0)	47 (8.5)	.15	0.039
Smombie	46 (5.9)	40 (7.2)	.39	0.024

**Table 8:** Influence of gender on smartphone usage behavior in older people (n=524, globally *P*=.07, V=0.13).

Behavior	Females, n=265, n (%)	Males, n=259, n (%)	*P* value	Cramér’s V
Not visible	249 (94.0)	229 (88.4)	.04	0.091
Talking	5 (1.9)	5 (1.9)	>.99	0.000
Headphones	0 (0)	4 (1.6)	.13	0.067
Holding	9 (3.4)	14 (5.4)	.36	0.040
Smombie	2 (0.7)	7 (2.7)	.17	0.060

### Effect of Pokemon Go Community Day

Our results showed that smombie behavior was clearly more frequent during the *Pokemon Go Community Day*. Table 9 shows the data distribution for this particular observation session. The proportion of smombies was 15.5% (21/136) on the *Pokemon Go Community Day* compared to 10.9% (361/3301) on normal days. Among teenagers, the proportion of smombies reached an impressive 61% (8/13) on the *Pokemon Go Community Day* compared to 14.6% (38/260) on normal days. This effect was also relevant in the young age range wherein the proportion of smombies reached 39% (12/31) during the *Pokemon Go Community Day* compared to 19.3% (228/1179) on the normal days. As expected, the chi-squared test showed that the effect was only statistically significant for teenagers (*P*<.001, V=0.244) and young people (*P*=.01, V=0.07).

**Table 9:** Demographic characteristics of the observed pedestrians on the Pokemon Go Community Day (N=136).

Characteristics		Value	
**Gender, n (%)**			
	Male		60 (44.1)
	Female		76 (55.9)
**Age (years), n (%)**			
	Teenagers (10-18 years)		13 (9.6)
	Young people (18-35 years)		31 (22.8)
	Adults (35-65 years)		65 (47.8)
	Older people (more than 65 years)		27 (19.9)
**Smartphone use, n (%)**			
	Not visible		105 (77.2)
	Talking		5 (3.7)
	Headphones		1 (0.7)
	Holding		4 (2.9)
	Smombie		21 (15.5)
**Scenarios, n (%)**			
	City center		136 (100)
	Residential areas		0 (0)
	University campus		0 (0)

Table 10 shows the global influence of *Pokemon Go CommunityDay* on the smartphone usage behavior.

**Figure 10:**
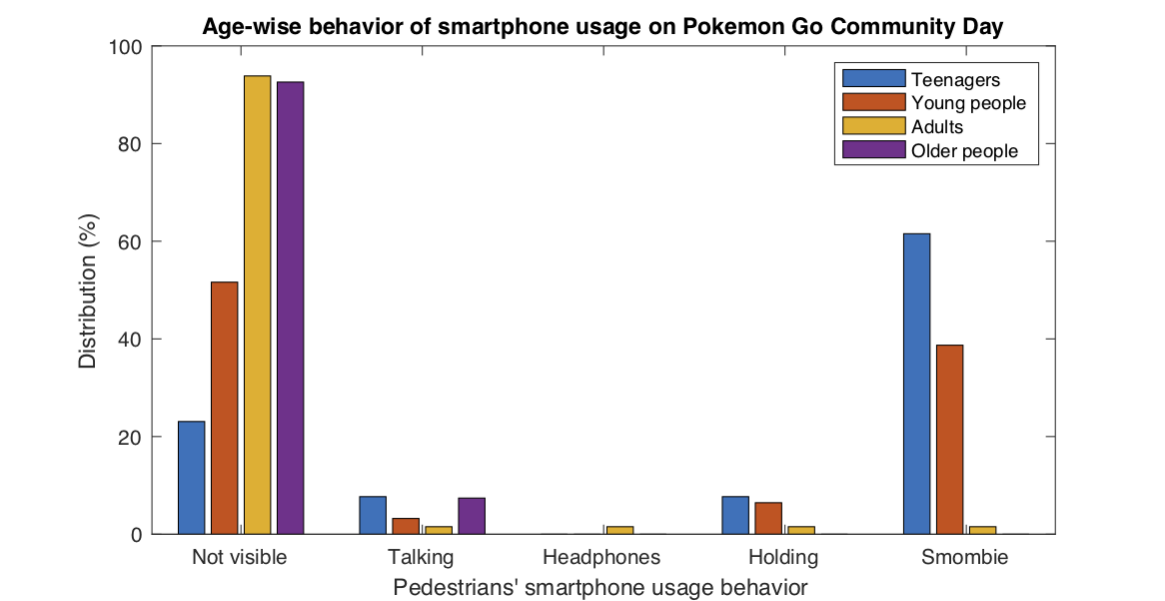
Smartphone usage behavior of pedestrians across different age ranges on the Pokemon Go Community Day. This figure shows graphically the observational results (%) from Tables 11-14.

Tables 11-14 show the influence of the *Pokemon Go CommunityDay* on the smartphone usage behavior in individuals of each age range. For a clearer interpretation, Figure 10 shows graphically the same results.

**Table 10:** Influence of the Pokemon Go Community Day on the smartphone usage behavior (globally *P*=.006, V=0.065).

Behavior	Normal day, N=3301, n (%)	Pokemon day, N=136, n (%)	*P* value	Cramér’s V
Not visible	2319 (70.3)	105 (77.2)	.10	0.028
Talking	141 (4.3)	5 (3.7)	.90	0.002
Headphones	99 (3.0)	1 (0.7)	.20	0.021
Holding	381 (11.5)	4 (2.9)	.003	0.051
Smombie	361 (10.9)	21 (15.5)	.13	0.025

**Table 11:** Influence of the Pokemon Go Community Day on the smartphone usage behavior in teenagers (globally *P*<.001, V=0.283).

Behavior	Normal day, n=260, n (%)	Pokemon day, n=13, n (%)	*P* value	Cramér’s V
Not visible	157 (60.4)	3 (23)	.02	0.144
Talking	6 (2.3)	1 (8)	.76	0.018
Headphones	16 (6.2)	0 (0)	.75	0.019
Holding	43 (16.5)	1 (8)	.64	0.028
Smombie	38 (14.6)	8 (61)	<.001	0.244

**Table 12:** Influence of the Pokemon Go Community Day on the smartphone usage behavior in young people (globally *P*=.06, V=0.087)

Behavior	Normal day, n=1179, n (%)	Pokemon day, n=31, n (%)	*P* value	Cramér’s V
Not visible	647 (54.9)	16 (52)	.86	0.005
Talking	67 (5.7)	1 (3)	.85	0.005
Headphones	55 (4.7)	0 (0)	.43	0.023
Holding	182 (15.4)	2 (6)	.26	0.032
Smombie	228 (19.3)	12 (39)	.01	0.070

**Table 13:** Influence of the Pokemon Go Community Day on the smartphone usage behavior in adults (globally *P*=.04, V=0.085).

Behavior	Normal day, n=1338, n (%)	Pokemon day, n=65, n (%)	*P* value	Cramér’s V
Not visible	1037 (77.5)	61 (94)	.003	0.079
Talking	58 (4.3)	1 (1)	.43	0.021
Headphones	24 (1.8)	1 (1)	>.99	0
Holding	133 (10.0)	1 (1)	.04	0.054
Smombie	86 (6.4)	1 (1)	.18	0.036

**Table 14:** Influence of the Pokemon Go Community Day on the smartphone usage behavior in older people (globally *P*=.25, V=0.099).

Behavior	Normal day, n=524, n (%)	Pokemon day, n=27, n (%)	*P* value	Cramér’s V
Not visible	478 (91.2)	25 (93)	>.99	0
Talking	10 (1.9)	2 (7)	.22	0.053
Headphones	4 (0.8)	0 (0)	>.99	0
Holding	23 (4.4)	0 (0)	.54	0.026
Smombie	9 (1.7)	0 (0)	>.99	0

## Discussion

### Principal Results

According to the results obtained, the incidence of smartphone usage among pedestrians was high, with almost one-third of the observed pedestrians belonging to “talking,” “headphones,” “holding,” or “smombie” categories. All these behaviors represent serious attention loss while walking. The most extreme situation, “smombie,” was observed in 1 of every 10 pedestrians. Regarding age groups, the data clearly show that young people are more likely to have smombie-like behavior. Considering gender, almost half of the young women observed showed “holding” or “smombie” behavior, and they comprised the highest proportion among all the age and gender groups.

The use of smartphones while walking on the street, including smombie behavior, should be analyzed from a cognitive perspective. It is known that the human information processing capacity is limited but learning and practice make it possible to automate many of the usual daily behaviors. These behaviors are characterized because they are developed with extensive practice, performed smoothly and efficiently, are resistant to modification, “unaffected” by other activities, do not interfere with other activities, and do not require mental effort [22]. A clear example of this behavioral automation is walking.

The automation of actions such as walking releases attentional resources that can be used to perform other tasks simultaneously. Human beings can divide their attention between different tasks simultaneously and execute them successfully as long as the attentional demands of these tasks do not exceed their attentional capacity. Otherwise, the cognitive system is overloaded and the performance decreases [23]. In practice, this implies that the processing of stimuli and the emission of responses can be done both automatically through a process of memory retrieval and in a conscious and controlled way [22]. These 2 mechanisms are activated for habitual behavior depending on the level of mastery of the task and the situation. If we are walking down the street, we do not require full awareness of how we should move our feet, legs, or arms and we can perform the walking behavior while thinking about our next task or watching the traffic. However, when a new stimulus arises, the behavior moves from automatic control to a more conscious level where we make some decision, for example, to stop. Attention can be consciously and voluntarily controlled and focused on a particular stimulus, but it can also be unconsciously captured by an external stimulus such as a loud noise [23]. The latter is what happens when a smombie stops on the road when he hears the horn of a car that is about to hit him.

The immersion of technology in people’s daily lives has enhanced the multitasking operation mode. The concept of media multitasking is defined as “engaging in one medium along with other media or nonmedia activities” [24]. Smombie behavior could fit within this definition. Multitasking implies the absence of total automation of tasks [23]. While it is true that walking and typing on the smartphone are fully automated activities for much of the population, identifying and avoiding obstacles or crafting meaningful messages requires active and controlled information processing. Studies on the influence of multitasking media in the educational environment show a significant reduction in student performance [23]. These results applied to the smombie phenomenon would explain the slowing down of walking, erratic wandering, and the increased risk of accidents (falls, running over, etc) [25].

The reduction of performance in multitasking media situations can not only be explained from the cognitive approach but should also be considered from the postphenomenological perspective that puts the focus on embodied habits and technical mediation in body-technology interaction [23]. For example, driving is more affected when talking on a hands-free phone than when talking to a codriver [26]. Similarly, the comprehension of information received during a lecture is greater when taking longhand notes than when writing on a computer. This is explained by the acquired modes of interaction with technology. Thus, when we write on computer keyboards, we tend to transcribe the words heard automatically, while when we take notes with pencil and paper, we are forced to reinterpret and synthesize the information perceived [23,27].

In summary, although behaving like a smombie sometimes goes well, it entails significant risks that, in addition to reducing performance in tasks that are carried out simultaneously, put the physical integrity of the individual at risk. Decreased attention, reduced peripheral vision when looking at the smartphone, and the activation of embodied habits in the interaction with technology are the main factors underlying the risk associated with the smombie phenomenon.

According to our results, the “holding” behavior was particularly common among young women. Further analyses are needed to clarify the reasons for this effect. Possible reasons include the lack of pockets in clothes, the social pressure to be available, theft prevention, psychological dependence, or for display of the smartphones as status symbols. We are currently designing survey-based experiments to gain insight into this effect.

When gender effect was analyzed specifically for each age range, it turned out that the higher difference between male and female behavior was found in old and young age ranges, while in teenagers and adults, the effect was not statistically significant. The main reason for gender differences in the old age range (older than 65 years) can be found in the highly differentiated roles of Spanish men and women in such a population. Concerning the young age range, the main reason may be due to the different use each gender makes of smartphones—young women are more attracted to social networks, while young men are more attracted to video games. Social networks urge the user to be ready for notifications and this may justify the high proportion of young women showing the “holding” behavior.

The extremely high proportion of smombies among teenagers during the *Pokemon Go Community Days* (8/13, 61%) suggests the need for specific interventions on such days. As *Pokemon Go* players usually concentrate on certain city areas, traffic and pedestrian crossings should be specially monitored in these areas.

### Comparison With Previous Studies

Previous observational studies have shown quantitative values of the number of pedestrians who use the smartphone while walking. In 2005, the study of Bungum et al [28] in Las Vegas, Nevada, already confirmed that 5.7% of the observed pedestrians (N=866) crossed the street while wearing headphones or while conversing on the phone. Some recent studies such as the one carried out in 2013 in Seattle, Washington, found that 29.8% of the observed pedestrians (N=1102) showed a distracting activity such as talking on the phone, texting, or listening to music [29].

An observational study in Paris in 2018 on the concept of “phone walkers” [20] surprisingly found that there were more female than male “phone walkers” (33.3% females, 19.7% males; N=3038). The statistical data of Schaposnik and Unwin [20], which are higher than those observed in our study, may be due to the mean age of the observed pedestrians who were younger since their estimated mean age was 35 years and they did not even consider people older than 65 years. Besides, their observational sessions were performed both on working days as well as weekends.

The observational data of the work of Ropaka et al [19] in Athens, Greece, in 2019 with a sample population of 2280 people is similar to our findings but with lower problematic smartphone use values: 83.4% of the pedestrians were classified as nondistracted (comparable to our “not visible” category with 70.3%), 5.0% were classified as distracted talking (comparable to our “talking” category with 4.3%), 5.4% as distracted listening to music (comparable to our “headphones” category with 3%), and 6.2% as distracted texting (comparable to our “smombie” category with 10.9%). In the study of Ropaka et al [19], they did not consider the “holding” behavior; further, the age ranges considered in their study were similar to that reported in our study, and the data were extracted from video analysis unlike our observation sessions in real time.

The obvious physical risk to which smombies are exposed is a problem with a solution that can be multifactorial and complex. Some cities have already taken structural measures to address this problem, such as the installation of a system of beacons at the edge of the pavement that function as a traffic light for pedestrians in Spain and Germany, the creation of phone lanes for smombies in China, the broadcasting of verbal messages on the railway in Hong Kong, or the writing of warning messages on the road (“Stop-Look-Cross. Answer Later” or “Heads Up, Phones Down”) [30-32]. However, the positive effects of some of these interventions seem to be diluted after the novelty period [33]. There are also technology initiatives such as Smombie Guardian, which is a smartphone app that uses the device’s camera to detect obstacles and alerts smombies through a red border and a vibration first and then an image of the obstacle with a colored border to prevent potential collisions [34].

### Limitations

The categorization of pedestrian behavior is not universal. In our study, we considered 5 categories of people with their smartphones: smartphone not visible, talking on smartphone, using headphones, holding a smartphone, and smombie. A different categorization may yield different results. Although previous studies by other researchers used a variety of different categorizations [13,35-41], this categorization was based on recent studies by Ropaka et al [19] and Schaposnik and Unwin [20]. Our study adopted Ropaka’s categories (not visible, talking, headphones, and smombie) and we added an extra category from the study of Schaposnik and Unwin (ie, holding or “phone walker”). Combined behaviors were not accounted for, for example, smombies who also wear headphones; such behaviors may cause a higher attention loss and may need specific consideration. Behaviors were ordered according to the increasing problematic smartphone use. Our ordering of problematic smartphone use is not universal and is possibly arguable. However, a different ordering would not affect the statistical results obtained. As stated in the Methods section, pedestrians checking their watches are not accounted for, because it was impossible to determine whether they were wearing a watch or a smartwatch. Not accounting for smartwatch smombies introduced a bias in the measures; the actual proportion of the smombies may be higher than that accounted for. Age categorization did not reflect the exact age of each pedestrian. Besides, observers could make wrong estimations of the age ranges. However, the kappa analysis performed comparing the age estimations of 2 different observers yielded correct results (=0.703; *P*<.001). Perhaps, the method used for data collection (the quick annotation app) can be somewhat dystopian because when the observer was walking as the pedestrians do, he or she was just a smombie counting other smombies. Therefore, the typical distractions of the smartphone (notifications and calls) together with having to coordinate their movements with the observation of pedestrians could produce errors in data collection. Data were gathered in a particular Spanish city from April to November 2019 only during weekdays (except for the *Pokemon Go Community Day*) and only during rush hours; therefore, the results obtained are restricted to such circumstances and may not extrapolate correctly to reflect global pedestrian smartphone usage. For example, the results may have been different during the winter months or in cities with a different culture or wealth status. Given the quick evolution of smartphone technology and user behavior, this study represents a snapshot of pedestrians’ smartphone usage in late 2019. Future studies are required to verify the results and analyze the trends in this topic. On the *Pokemon Go Community Day*, the study only covered 136 pedestrians; therefore, more data should be registered to confirm our results.

### Future Research

A modified version of the quick annotation app used for the experiment is currently under development. The goal is to perform quick surveys and gather extra information. We are interested in determining why the “holding” behavior is increasingly common, particularly among young women. The survey will offer several answers to the question: why are you carrying your smartphone in your hand? Future work also includes an observational study of indoor walking while using the smartphone. The goal is to analyze the behavior of employees with their smartphones when they are moving around in office environments through the aisles and stairs.

### Implications

Different actions can be carried out. First, an extra educational effort needs to be taken to raise the awareness about the risks of using the smartphone while walking. Second, cities need to be redesigned, thereby making them safer for smartphone users, by creating specific lanes and adding visual and sound signals in street crossings. Third, smartphones should be developed with prevention tools. Among these prevention tools, the simplest ones may just warn their users when the device is being used while the smartphone sensors detect the walking activity. More complex tools can alert the user when an obstacle is detected and a collision is imminent. Finally, other tools may be capable of blocking highly distractive apps while the user is walking.

### Conclusions

The incidence of smartphone usage among pedestrians is high. Our study registered almost one-third of the pedestrians interacting with the smartphone in different ways, and more than 1 of each 10 pedestrians behaving as a smombie. According to the data gathered, the groups of greatest risk and, therefore, the groups that the interventions should be directed to, are the groups of adolescents and young people.

Besides, this study offers quantitative data about an increasingly common behavior with the smartphone—holding it while walking or, in other words, keeping it in the hand to immediately respond to any notification. According to the data collected, this behavior is more common in females, particularly among female adolescents and young women.

## Appendix

Multimedia Appendix 1 Confusion matrices obtained in the Cohen kappa analysis for each category of the study.
