# Supplementary material for: Factors Influencing the Smartphone Usage Behavior of Pedestrians: Observational Study on “Spanish Smombies”
Source: J Med Internet Res. 2020 Aug 14;22(8):e19350. doi: 10.2196/19350 (PMC7455880; doi:10.2196/19350)
Supplement: Multimedia Appendix 1 [file jmir_v22i8e19350_app1.docx]

**Multimedia Appendix 1. *Confusion matrices obtained in the Cohen kappa analysis for each category of the study.***

The following matrices are the ***confusion matrices*** obtained in the Cohen kappa analysis for each category of the study:

| GENDER | Female | Male |
| --- | --- | --- |
| Female | 58 | 0 |
| Male | 0 | 42 |

$$\begin{matrix} female & 58 & 0 \\ male & 0 & 42 \end{matrix}$$

| AGE | Teenagers | Young people | Adults | Older people |
| --- | --- | --- | --- | --- |
| Teenagers | 6 | 4 | 0 | 0 |
| Young people | 1 | 33 | 9 | 0 |
| Adults | 0 | 3 | 35 | 2 |
| Older people | 0 | 0 | 0 | 7 |

$$\begin{matrix} AGE & teen & young & adult & old \\ teen & 6 & 4 & 0 & 0 \\ young & 1 & 33 & 9 & 0 \\ adult & 0 & 3 & 35 & 2 \\ old & 0 & 0 & 0 & 7 \end{matrix}$$

| BEHAVIOR | Not visible | Talking | Headphones | Smombie | Smombie |
| --- | --- | --- | --- | --- | --- |
| Not visible | 52 | 0 | 0 | 1 | 0 |
| Talking | 0 | 8 | 0 | 0 | 0 |
| Headphones | 0 | 0 | 2 | 0 | 0 |
| Smombie | 0 | 0 | 0 | 13 | 2 |
| Smombie | 0 | 0 | 0 | 0 | 22 |

$$\begin{matrix} BEHAVIOUR & without & talking & music & on hand & smombie \\ without & 52 & 0 & 0 & 1 & 0 \\ talking & 0 & 8 & 0 & 0 & 0 \\ music & 0 & 0 & 2 & 0 & 0 \\ on hand & 0 & 0 & 0 & 13 & 2 \\ smombie & 0 & 0 & 0 & 0 & 22 \end{matrix}$$
